# New Insights on Volatile Components of *Vanilla planifolia* Cultivated in Taiwan

**DOI:** 10.3390/molecules26123608

**Published:** 2021-06-12

**Authors:** Chih-Hsin Yeh, Kai-Yi Chen, Chia-Yi Chou, Hsin-Yi Liao, Hsin-Chun Chen

**Affiliations:** 1 Taoyuan District Agricultural Research and Extension Station, Council of Agriculture, Executive Yuan, Taoyuan 327, Taiwan; zeamays@tydais.gov.tw (C.-H.Y.); white981981@gmail.com (C.-Y.C.); 2 Department of Agronomy, National Taiwan University, Taipei 106, Taiwan; kaiychen@ntu.edu.tw; 3 Department of Cosmeceutics, China Medical University, Taichung 406, Taiwan

**Keywords:** vanilla, GC-FID, headspace solid-phase microextraction (HS-SPME), volatile components

## Abstract

Vanilla (*Vanilla planifolia*) is a precious natural flavoring that is commonly used throughout the world. In the past, all vanilla used in Taiwan was imported; however, recent breakthroughs in cultivation and processing technology have allowed Taiwan to produce its own supply of vanilla. In this study, headspace solid-phase microextraction (HS-SPME) combined with GC-FID and GC-MS was used to analyze the volatile components of vanilla from different origins produced in Taiwan under different cultivation and processing conditions. The results of our study revealed that when comparing different harvest maturities, the composition diversity and total volatile content were both higher when the pods were matured for more than 38 weeks. When comparing different killing conditions, we observed that the highest vanillin percentage was present after vanilla pods were killed three times in 65 °C treatments for 1 min each. From the experiment examining the addition of different strains, the PCA results revealed that the volatiles of vanilla that was processed with *Dekkera bruxellensis* and *Bacillus subtili*s was clearly distinguished from which obtained by processing with the other strains. Vanilla processed with *B. subtilis* contained 2-ethyl-1-hexanol, and this was not detected in other vanillas. Finally, when comparing the vanillin percentage from seven different regions in Taiwan, vanilla percentage from Taitung and Taoyuan Longtan were the highest.

## 1. Introduction

Vanilla plays an important role in food flavoring. This compound is derived after curing from the fruit pods of *Vanilla* spp. and is widely used in the food, beverage, perfume, and pharmaceutical industries [1]. *Vanilla* spp. belongs to the Orchidaceae *Vanilla* that is a perennial vine and evergreen plant, which is the only edible spice crop of the family [2]. It is native to Central and South America and widely cultivated in tropical regions, as in Madagascar (80%) [3]. The *Vanilla* Swartz genus has more than 100 species, amongst which 15 are aromatic. Among the 110 species of vanilla known today, *Vanilla planifolia*, *Vanilla tahitensis*, and *Vanilla pompona* are widely used commercially [4,5].

The fresh pods of vanilla do not possess the common vanilla smell of well-known food products. Their unique aroma appears after the curing process. The processing steps typically vary among countries and regions; however, these steps are predominantly performed in accordance with the method described by Ramachandra Rao and Ravishankar [5], who described four stages of vanilla curing including killing, sweating, drying, and conditioning. As every vanilla pod must be artificially pollinated and the process is complicated, vanilla is the second most expensive edible spice in the world, behind only saffron [2,6].

The use of natural flavors throughout the world is becoming increasingly common. Vanilla is the flavoring most widely used worldwide, occupying a prominent place in the market. As an important food condiment, the demand for vanilla has also increased significantly [7,8]. In 2018, the cost of vanilla was 600 US dollars per kilogram. In 2020, the price of vanilla pods in the international market was approximately 250–350 US dollars per kilogram (including 1–2% vanillin), while the price of synthetic vanillin was only 10 US dollars per kilogram [9]. Therefore, it remains difficult to substantially replace the artificial vanilla extract. Simultaneously, the demand for vanilla pods in Taiwan has increased. In 2007, Taoyuan District Agricultural Research and Extension Station, Council of Agriculture, Executive Yuan (hereinafter referred to as Taoyuan Station) successfully produced vanilla pods based on a breakthrough in vanilla cultivation technology. Since 2017, due to the technological achievements of agriculture, locally cultivated vanilla has been directly produced and purchased in Taiwan.

The vanilla industry in Taiwan remains at an early stage of development. The differences in climate, cultivation, and processing methods differ from those in foreign countries. Studies have shown that the aroma characteristics of vanilla not only depend on its own genetic characteristics but also vary across countries. The unique climate, growth conditions, and technology in terms of processing methods and storage conditions are different [10,11]. Therefore, understanding the differences in aroma components is a primary issue.

The purpose of this study was to tentatively identify if Taiwan vanilla possesses unique aroma components and to explore the variations in its components and relative percentage under different processing conditions during cultivation and curing. We hope that the results of this study will tentatively identify the optimal processing conditions to contribute to the future development of the vanilla industry in Taiwan.

## 2. Results

### 2.1. Analysis of the Volatile Components of Taiwan-Cultivated Vanilla Pods Using HS-SPME

The results tentatively identified 31 volatile components (Table 1), including one monoterpene, two ketones, three esters, four phenols, five alcohols, five sesquiterpenes, nine aldehydes, and two others, and the aldehydes were primarily responsible for the volatiles of the vanilla pods.

The primary component of vanilla cultivated in Taiwan is vanillin (73.85%), and this compound is an aldehyde. This compound has been previously identified in the literature as exhibiting a sweet smell that is reminiscent of vanilla [12]. The second is guaiacol (2.01%) that was identified by GC-O as a smoky vanilla pod possessing a phenolic smell [13]. The relative percentage of the remaining volatile components did not exceed 1%.

Hassan et al. [14] analyzed Madagascar vanilla pods that were ground into powder after cold storage using HS-SPME. A total of 77 volatile components were identified, and of these, 18 were also tentatively identified in this study. The most important component was vanillin (48.02%). Additionally, the five sesquiterpenes mentioned in the study as being identified in *V. planifolia* for the first time were also tentatively identified in our study. They contributed to the vanilla aroma of citrus, lemon, wood, or freshness. Liang et al. [15] also analyzed vanilla pod powder using HS-SPME, and they identified 28 volatile components. The major and minor components tentatively identified in their study were the same as those identified in our experiment (vanillin and guaiacol). Other similarly tentatively identified components included furfural, benzaldehyde, benzyl alcohol, and methyl salicylate.

### 2.2. Comparison of Volatile Components under Different Conditions during the Processing of Taiwan-Cultivated Vanilla

#### 2.2.1. Different Harvest Maturity

The vanilla pods can be harvested approximately 38 weeks after flower pollination and then further processed. To ensure the quality of vanilla pods, we compared the volatile components of vanilla pods possessing different harvest maturities that were harvested at 34, 38, and 42 weeks after pollination.

A total of 23 volatile components were tentatively identified in the vanillas cultivated in Taiwan that possessed different harvest maturities (Table 2). Vanilla pods with less than 38 weeks of maturation possessed less volatile components, while vanilla pods matured for 42 weeks contained increased levels of 3-octen-2-one, 1-octanol, and phenylethyl alcohol. Xu et al. [16] noted that the aroma of 3-octen-2-one contributes to the earthy smell and phenylethyl alcohol possesses a floral scent or a rose aroma [13]. However, the percentage of these components were not high. Based on the results, the vanilla pods that were matured for more than 38 weeks were relatively high in volatile components, regardless of the component diversity or total volatile content. Therefore, it is recommended that the vanilla pods be harvested at 38 weeks after pollination to ensure optimal harvested maturity.

#### 2.2.2. Different Conditions during the Curing Process

Current studies have determined that volatile components can be released during the killing and sweating stages of the curing process and adjusting the process can promote the generation of volatile components [17]. Mariezcurrena et al. [18] focused on the killing stage that destroys the plant cell wall through the use of hot-water immersion or freezing at different temperatures. It is important to quickly and effectively destroy the cell structure without damaging the enzymes to allow for the generation of volatile components. Our current study refers to Mariezcurrena et al. [18] and modified its methods by designing four different killing temperatures and time conditions (vanilla pod immersion in hot water at 65 °C three times for 1 min each, 65 °C for 3 min, 70 °C three times for 30 s each, and 80 °C three times for 10 s each) and two different fermentation temperatures (40 °C and 45 °C) for vanilla cultivated in Taiwan. We compared the volatiles of the vanilla pods, and all of the components and relative percentage of the different curing conditions are shown in Table 3. The main component of all extracts was vanillin.

The first step of the vanilla curing process is killing, and this step aims to promote the reaction of enzymes by destroying the cell structure and halting the further aging of fresh pods [19]. According to the results presented in Table 3, the four different killing methods result in similar component quantities. Only a small number of components are present in very small amounts, and these have not yet been identified. A more obvious difference was observed for vanillin. The highest percentage (79%) was obtained by killing using three treatments at 65 °C for 1 min each, and the lowest percentage (62%) was observed in pods that were killed using three treatments at 80 °C for 10 s each. When comparing the relative percentage of the main and minor components of vanilla pods obtained using four different killing temperature methods (Table 3), it was observed that the content of vanillin gradually decreased when immersed in hot water for a short time at high temperature and that the other minor components, including benzyl alcohol, guaiacol, and *p*-creosol, gradually increased after a short period of high-temperature treatment.

The second step of the vanilla curing process is sweating, and two sweating temperatures were compared. According to the results presented in Table 3, the two different temperatures did not exert any different effects on aroma. Only a small number of trace components were tentatively identified in vanilla pods at 45 °C, while sweating at 40 °C resulted in higher levels of phenolic compounds such as *p*-cresol, guaiacol, and *p*-creosol.

#### 2.2.3. Microorganism Treatments during the Curing Process

When examining the production of vanilla flavor, studies have reported that plant enzymes and microbial activities influence the aroma of natural vanilla [20]. It was found that enzymatic hydrolysis occurred on the surface of vanilla beans [21]. For example, Gu et al. [22] determined that *Bacillus* participates in the hydrolysis of glucovanillin during curing and related to vanillin formation during conventional curing. Based on these studies, we added various strains to vanilla pods during the curing process in an attempt to observe their effect on the volatile components. The added strains included three different yeasts (*D. bruxellensis* [Y1], *S. crataegensis* [Y2], and *C. cacaoi* [Y3]) and *B. subtilis* (B), all of which were compared to the control group.

A total of 35 volatile components were tentatively identified in this study. Table 4 lists the components and relative percentage of the vanilla pods supplemented with different strains. The main component of all the samples was vanillin (66–83%). When comparing the volatile components of vanilla pods in the control group and different yeast strains, we determined that the percentage of the minor components in the yeast-treated groups was higher than that in the control, particularly for benzyl alcohol and *p*-cresol. The percentage of the secondary components of B was relatively low, and guaiacol and benzyl alcohol were typically the most prevalent. However, 2-ethyl-1-hexanol, a compound that possesses a delicate and floral fragrance, has not been previously detected in other species [23].

When comparing vanilla pods that were treated with different strains, we observed that there was no significant aroma change among the groups. The other important ingredients, with the exception of vanillin, are selected including furfural, benzyl alcohol, *p*-cresol, guaiacol and *p*-creosol for comparison (Table 4). The results revealed that among the three groups supplemented with yeast, all minor components were the highest in Y1. This strain has received attention in the food industry and academic research in recent years due to its characteristic smell and its frequent use for fermentation of different wines [24]. Our results revealed that the fermentation of this strain causes the vanilla pods to possess a richer odor that affects the volatile components of vanilla.

The percentage of benzyl alcohol in Y2 was highest and resulted in floral and sweet aromas [25]. The minor components of the vanilla pods in the control group were low, with the exception of guaiacol. These results are the same as those for natural vanilla pods sold abroad, and the main components of those pods are vanillin and guaiacol [26]. The percentage of minor components in sample B was low, particularly for guaiacol. Based on this, we speculated that the addition of *B. subtilis* to vanilla pods results in a weaker smoky smell. Our findings confirm that the addition of microorganisms to vanilla pods affects their flavor [20].

**Figure 1:**
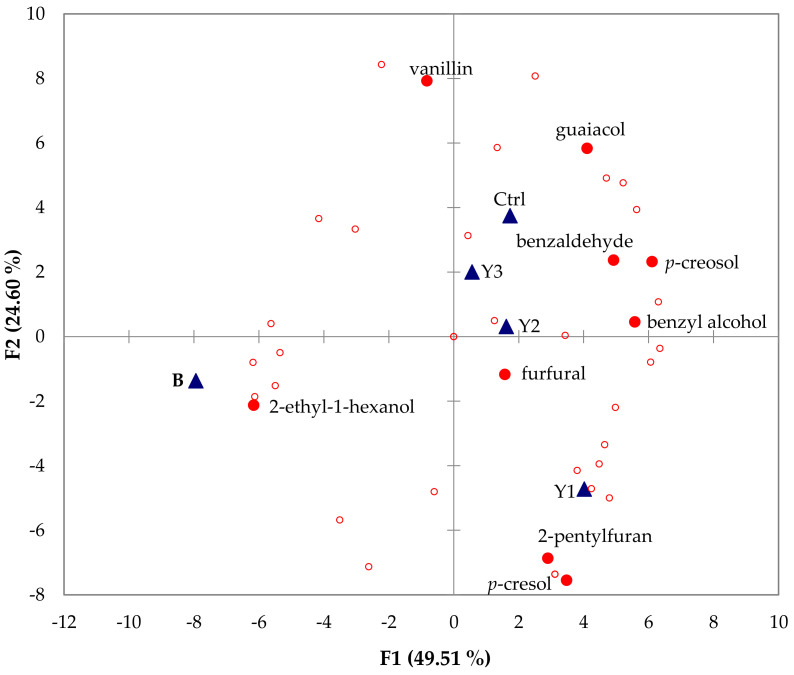
Principal component analysis diagram of vanilla pods supplemented with different microorganism treatments. ▲: Samples (ctrl: Standard procedure; Y1: *D. bruxellensis*; Y2: *S. crataegensis*; Y3: *C. cacaoi*; B: *B. subtilis*). ●: Labeled volatile compounds. ○: Unlabeled volatile compounds.

To better understand the effects of different strains on the volatiles of vanilla pods, we performed principal component analysis (PCA) to observe the correlation between volatile components and samples (Figure 1). All component data in PCA was from Table 4. The statistical results divided the samples into three groups, primarily distinguishing the vanilla samples of *B. subtilis* and *D. bruxellensis*. In regard to vanillin, there was not much diversity among all these groups, and the only obvious difference was for Y1.

The percentage of vanillin in all samples was relatively high, thus suggesting that this compound was not an indicator component of any group and did not affect the classification of the samples. The component closest to B is 2-ethyl-1-hexanol, thus indicating that it is related to the addition of *B. subtilis*. The components closest to the Y1 sample are *p*-cresol and 2-pentylfuran, both of which exhibit lower percentage levels in the other vanilla samples. Using PCA, we inferred that the percentage of these two components was increased in response to the addition of *D. bruxellensis*. The four components that were closer to the control, Y2, and Y3 groups were guaiacol, furfural, benzaldehyde, benzyl alcohol, and *p*-creosol, thus indicating that these four components are not affected by the addition of *B. subtilis* or *D. bruxellensis*. Based on the statistical results presented above, we can infer that the effects of *S. crataegensis* and *C. cacaoi* on vanilla are similar to those exerted by the microbial metabolic pathways of natural vanilla pods and that they belong to the same group as the control group. Vanilla pods processed with *D. bruxellensis* and *B. subtilis* exhibit different aromas due to the different metabolic pathways of these organisms, and this is consistent with the results of previous studies. The addition of this strain during the vanilla pod curing process produces β-glucosidase, a compound that affects flavor formation [21].

### 2.3. Comparison of Volatile Components of Taiwan-Cultivated Vanilla from Different Regions

It has been previously determined that the characteristics of vanilla depend not only on genetic characteristics but also on the unique climate, cultivation conditions, and technical methods of each country or region [11]. Therefore, differences in the volatiles of vanilla pods of different origins have also been discussed in recent years. Januszewska et al. [27] speculated that vanilla aroma would be affected by geographic origin and may then indirectly affect other vanilla products such as chocolate or milk. Sensory analysis also confirmed that the vanilla pods from different regions were different.

The experiments in our study were conducted at Taoyuan Station on pods obtained from seven other regions in Taiwan (Taoyuan Xinwu, Longtan, Hsinchu Xinpu, Nantou Mingjian, Yunlin Sihu, Taitung Station, and Taoyuan Station), and we compared the volatile components of vanilla pods obtained from these different regions. The volatile components and their relative percentage are listed in Table 5. The difference in the content is clear in regard to vanillin. The samples from Taitung Station and Taoyuan Longtan possessed higher amounts of vanillin (83%), and the vanillin percentage of samples from Nantou Mingjian was lower (67%). When comparing the total volatile contents and primary component content of vanilla cultivated in Taiwan among different regions, we observed that the percentage of vanillin was highest in pods from Taitung Station and Taoyuan Longtan. This indicates that the total volatile content was affected by vanillin. However, differences still remain due to variations in the other components.

## 3. Materials and Methods

### 3.1. Plant Materials

All vanilla samples were *V. planifolia* and were cultivated by Taoyuan Station, Taitung District Agricultural Research and Extension Station (hereinafter referred to as Taitung Station), and by domestic farmers. Subsequently, all samples were sent to Taoyuan Station for processing, and the samples are all listed in Table 6. The usual killing temperature was 65 °C for 3 min. When it reached the sweating stage, all pods were humidified at the ambient temperature of 40–45 °C for 2 days, and were 10 days under a variable temperature environment of 35–40 °C. Different condition tests were subject to the test treatment. Finally, after sweating, the pods were dried at 25 °C. When the pods were dried to about 35–38% moisture content, they were transferred to wooden boxes for 3 months of conditioning.

### 3.2. Different Vanilla Processing Conditions

#### 3.2.1. Comparison of Volatile Components under Different Conditions during the Processing of Taiwan-Cultivated Vanilla

This study sought to explore if the volatile components of vanilla pods differed according to their harvest maturity and processing conditions:

(1) Different harvest maturity tests.

More than eight months was required for vanilla pods to mature in Taiwan (the time from pollination to harvest of the pods). To test different harvest maturities, vanilla pods that were pollinated at 34, 38, and 42 weeks prior to harvest were prepared.

(2) Different curing conditions.

There are four stages of vanilla pod curing that include killing, sweating, drying, and conditioning. The Taoyuan Station used a fermentation machine (Metro C5 1 Series Holding/Proofing Cabinet, Wilkes-Barre, PA, USA) to replace the sweating and drying stages that require natural sunlight in traditional processes. The fermentation machine can better control the temperature and humidity to save processing time and provide improved quality control. This study conducted temperature adjustments for volatile components analysis at the following different curing stages:

(a) Killing temperature and time: fresh vanilla pods were tested under the following four conditions: vanilla pods immersion in hot water at 65 °C three times for 1 min each, 65 °C for 3 min, 70 °C three times for 30 s each, and 80 °C three times for 10 s each.
(b) Sweating temperature: the vanilla pods were subjected to sweating at 40 °C and 45 °C in the fermentation machine after killing. The pods were treated at 40 or 45 °C for 8 h, then moved to room temperature for 16 h and temperature-changed for 10 days.

(3) Microorganism treatment during the curing process

Vanilla pods were treated with three different yeast strains (*D. bruxellensis*, *S. crataegensis*, and *C. cacaoi*) and a bacterial strain (*B. subtilis*) to compared the volatiles. The process to yeast treatment: while water content of the pods was closed to 38% during the drying stage, the pods were evenly attached to yeast solutions then air-dried. The concentration of yeast solutions was 1 × 10^6^–1 × 10^7^ cfu/mL. Vanilla pods were treated once every 7 days, a total of 3 times, and finally placed in wooden boxes for condition. The method of *Bacillus* treatment was: the pods were treated with the bacterial solution three times respectively after the killing stage, the sweating stage, and on the 14th day of drying. The concentration of the bacterial solution was 1 × 10^6^–1 × 10^7^ cfu/mL.

#### 3.2.2. Comparison of Volatile Components of Taiwan-Cultivated Vanilla from Different Regions

Taoyuan Station obtained samples from seven other regions in Taiwan (Taoyuan Xinwu, Longtan, Hsinchu Xinpu, Nantou Mingjian, Yunlin Sihu, Taitung Station, and Taoyuan Station) for comparison. The volatile components analysis comparison was performed using the above sample preparation method and HS-SPME.

### 3.3. HS-SPME Operating Conditions and GC / GC-MS Instrument Analysis

#### 3.3.1. Analysis of Vanilla Pods Using HS-SPME

The Taiwan cultivated vanilla pods were selected 8–12 pods with similar length and weight (about 30 g). The pods were cut in half. The seeds were scraped, and randomly took 1 g sample placed in a 4 mL transparent sample bottle. The bottle was then sealed (hole Cap PTEE/silicone septa). For SPME extraction, a 65 μm PDMS/DVB (Supelco, Bellefonte, PA, USA) coated fiber was used for the volatile extraction. The extraction temperature was 50 °C and the extraction time was 40 min. After extraction, the fiber was injected into the GC inlet for GC-FID and GC-MS analysis, and the volatile components were subsequently tentatively identified. All the experiments in this study were carried out in triplicate with both instrument analysis.

#### 3.3.2. Gas Chromatography (GC-FID)

The volatile compounds were analyzed using a 7890 A GC system (Agilent, Palo Alto, CA, USA) equipped with a 60 m × 0.25 mm i.d. DB-1 fused-silica capillary non-polar column with a film thickness of 0.25 μm and a flame ionization detector. The oven temperature was maintained at 40 °C for 1 min, then raised to 150 °C at 5 °C/min, held for 1 min, raised to 200 °C at 10 °C/min, and then maintained at this temperature for 11 min. The injector and detector temperatures were maintained at 250 °C and 300 °C, respectively. The carrier gas (nitrogen) flow rate was 1.0 mL/min. When HS-SPME was used as the method, the injection port was set to splitless.

#### 3.3.3. Gas Chromatography-Mass Spectrometry (GC-MS)

The volatile compounds were analyzed using an Agilent 7890 B GC coupled to an Agilent 5977A quadrupole mass spectrometer (MSD). The capillary column and GC-FID conditions for the GC-MS analysis were the same as those used in the GC analysis. The carrier gas (helium) flow rate was 1 mL/min. The electron energy was 70 eV at 230 °C, and the quadrupole temperature was 150 °C. The constituents were tentatively identified by matching their spectra to those recorded in an MS library (Wiley 7n). The retention index (RI) of the volatile components was based on standards of C_5_–C_25_ *n*-alkane mixture (Sigma-Aldrich Chem. Co., St. Louis, MO, USA), and basis was calculated by the method of Kovát [28].

#### 3.3.4. Relative Percentage Calculation

After volatile components were identified, the percentage composition was calculated using the peak area normalization measurements. The formula is as follows:

$$\frac{volatile\;component\;peak\;area}{total\;peak\;areas}\times 100\%$$

In addition to the volatile compounds of the sample, HS-SPME will also adsorb the impurity of the bottle or any silicon-containing coating. Total percentage in the above tables not reached 100%, due to deducted from these impurities.

### 3.4. Statistical Analysis

The data were subjected to one-way analysis of variance, with Tukey’s multiple range method used to identify significant differences of *p* < 0.05 with GraphPad Prism 5 (GraphPad Software, San Diego, CA, USA). Principal component analysis (PCA) was applied to the data set with XLSTAT 2014 (Addinsoft, New York, NY, USA).

## 4. Conclusions

A total of 31 volatile components have been tentatively identified in vanilla cultivated in Taiwan, and the main component was vanillin. When comparing vanilla pods possessing different harvest maturities, we determined that vanilla pods that were matured for more than 38 weeks possessed higher composition diversity and total volatile content. After comparing vanilla pods subjected to four different killing conditions, we observed that vanillin was present at the highest percentage t after killing by three treatments at 65 °C for 1 min each. There was no significant effect on the volatile components between the two sweating temperatures. In the experiment involving the addition of different microbial strains, the *B. subtilis* sample contained 2-ethyl-1-hexanol, and this was not detected in the other samples. The PCA results revealed that the volatile components of vanilla processed with *D. bruxellensis* and *B. subtilis* was clearly distinguished from that of the control. Finally, by comparing vanilla from seven different regions in Taiwan, a difference can be observed in regard to the percentage of vanillin.

## Figures and Tables

**Table 1:** Analysis of volatile compounds and relative percentage from Taiwan cultivated vanilla using HS-SPME.

Compounds ^A^	RI ^B^	Relative Percentage ^C^ (%)
***Alcohols***		
benzyl alcohol	999	0.77 ± 0.14
2-ethyl-1-hexanol	1007	<0.01
phenethyl alcohol	1080	0.29 ± 0.04
anise alcohol	1250	0.39 ± 0.02
cinnamic alcohol	1268	0.06 ± 0.00
***Aldehydes***		
furfural	799	0.56 ± 0.12
benzaldehyde	928	0.25 ± 0.05
salicylaldehyde	1006	0.22 ± 0.07
nonanal	1074	0.12 ± 0.02
safranal	1172	0.08 ± 0.02
anisaldehyde	1212	0.16 ± 0.01
*p*-hydroxybenzaldehyde	1335	1.32 ± 0.15
vanillin	1373	75.80 ± 9.64
methylvanillin	1428	0.20 ± 0.06
***Esters***		
benzyl acetate	1125	0.13 ± 0.01
methyl salicylate	1166	0.31 ± 0.09
phenethyl acetate	1221	0.06 ± 0.00
***Ketones***		
acetophenone	1030	<0.01
acetovanillone	1446	0.12 ± 0.00
***Monoterpene***		
limonene	1016	0.14 ± 0.03
*Phenols*		
*p*-cresol	1043	0.69 ± 0.12
guaiacol	1056	1.78 ± 0.30
*p*-creosol	1161	0.46 ± 0.06
*p*-vinylguaiacol	1280	0.14 ± 0.01
***Sesquiterpenes***		
α-copaene	1380	0.17 ± 0.03
α-bergamotene	1441	0.49 ± 0.04
α-amorphene	1474	0.08 ± 0.00
α-muurolene	1498	0.12 ± 0.05
δ-cadinene	1517	0.07 ± 0.01
***Others***		
3,5-octadien-2-one	1035	0.08 ± 0.04
tetramethylpyrazine	1061	0.06 ± 0.02
**unknown**		13.36
**total**		85.17

^A^ Tentatively identification of components based on GC-MS library (Wiley 7n). ^B^ Retention indices, using paraffin (C_5_-C_25_) as references. ^C^ Relative percentages from GC-FID, values are means ± SD of triplicates.

**Table 2:** Volatile compounds and their relative percentage in vanilla pods at different harvest maturities.

RI ^A^	Compounds ^B^	Relative Percentage ^C^ (%)		
		Different Maturity (Weeks)		
		34 ^D^	38 ^E^	42 ^F^
799	furfural	0.20 ± 0.10a	0.41 ± 0.20a	0.28 ± 0.10a
928	benzaldehyde	0.13 ± 0.03a	0.31 ± 0.20a	0.22 ± 0.11a
959	phenol	0.46 ± 0.10a	1.21 ± 0.87a	0.71 ± 0.37a
999	benzyl alcohol	0.83 ± 0.12a	0.74 ± 0.37a	1.16 ± 0.18a
1005	3-octen-2-one	-^G^	-	0.62 ± 0.50
1017	2-hydroxybenzaldehyde	0.08 ± 0.07a	0.09 ± 0.04a	0.17 ± 0.15a
1043	*p*-cresol	0.19 ± 0.04a	0.41 ± 0.22a	0.29 ± 0.07a
1049	1-octanol	-	-	0.06 ± 0.02
1056	guaiacol	1.42 ± 0.38a	2.03 ± 0.97a	2.43 ± 0.58a
1074	nonanal	1.27 ± 0.24a	0.29 ± 0.18b	0.91 ± 0.21a
1080	phenylethyl alcohol	-	0.22 ± 0.07	0.23 ± 0.04
1125	benzyl acetate	0.09 ± 0.01a	0.12 ± 0.02a	0.12 ± 0.03a
1161	*p*-creosol	0.24 ± 0.05a	0.38 ± 0.13a	0.37 ± 0.08a
1166	methyl salicylate	0.19 ± 0.05a	0.18 ± 0.08a	0.16 ± 0.05a
1180	safranal	0.13 ± 0.05a	0.06 ± 0.03a	0.08 ± 0.03a
1212	anisaldehyde	0.07 ± 0.01a	0.08 ± 0.02a	0.08 ± 0.02a
1221	phenethyl acetate	0.03 ± 0.01a	0.05 ± 0.02a	0.04 ± 0.02a
1250	anise alcohol	0.23 ± 0.02a	0.24 ± 0.04a	0.29 ± 0.02a
1268	cinnamyl alcohol	0.04 ± 0.01a	0.03 ± 0.00a	0.05 ± 0.01a
1280	*p*-vinylguaiacol	0.07 ± 0.00a	0.09 ± 0.02a	0.09 ± 0.02a
1335	*p*-hydroxybenzaldehyde	0.62 ± 0.08a	0.63 ± 0.03a	0.71 ± 0.08a
1373	vanillin	86.74 ± 3.01a	73.41 ± 2.32b	80.44 ± 4.60b
	total	93.06	80.96	89.50

^A^ Retention indices, using paraffin (C_5_–C_25_) as references. ^B^ Tentatively identification of components based on GC-MS library (Wiley 7n). ^C^ Relative percentages from GC-FID, values are means ± SD of triplicates. Different letters within the same line denote significant difference in Tukey’s multiple test (*p* < 0.05). ^D^ pollinated at 34weeks prior to harvest. ^E^ pollinated at 38weeks prior to harvest. ^F^ pollinated at 42weeks prior to harvest. ^G^ undectable.

**Table 3:** Volatile compounds and their relative percentage in vanilla pods under different processing conditions.

RI ^A^	Compounds ^B^	Relative Percentage ^C^ (%)					
		K1 ^E^	K2 ^F^	K3 ^G^	K4 ^H^	S1 ^I^	S2 ^J^
799	furfural	0.45 ± 0.04a	0.51 ± 0.02a	0.63 ± 0.10a	0.50 ± 0.08a	0.54 ± 0.05a	0.51 ± 0.16a
928	benzaldehyde	0.38 ± 0.16a	0.29 ± 0.03a	0.30 ± 0.04a	0.31 ± 0.04a	0.22 ± 0.04a	0.20 ± 0.08a
959	phenol	- ^D^	<0.01	-	-	<0.01	<0.01
999	benzyl alcohol	1.15 ± 0.17a	1.26 ± 0.13a	1.42 ± 0.22a	1.63 ± 0.21a	0.96 ± 0.12b	0.97 ± 0.20b
1007	2-ethyl-1-hexanol	0.23 ± 0.01	0.29 ± 0.02	<0.01	<0.01	<0.01	<0.01
1016	limonene	-	-	-	0.07 ± 0.01	<0.01	0.04 ± 0.01
1030	acetophenone	-	-	0.04 ± 0.00	0.07 ± 0.04	-	-
1043	*p*-cresol	0.91 ± 0.06a	1.10 ± 0.07a	1.08 ± 0.12a	0.80 ± 0.09b	0.72 ± 0.02b	0.67 ± 0.07b
1049	1-octanol	<0.01	<0.01	<0.01	-	<0.01	<0.01
1056	guaiacol	2.83 ± 0.12c	2.94 ± 0.17bc	4.31 ± 0.69b	6.89 ± 1.02a	1.80 ± 0.02cd	1.28 ± 0.09d
1061	tetramethylpyrazine	<0.01	<0.01	-	<0.01	0.08 ± 0.01	0.07 ± 0.01
1074	nonanal	0.09 ± 0.01b	0.10 ± 0.01ab	-	0.13 ± 0.02a	0.10 ± 0.01ab	0.08 ± 0.01b
1080	phenylethyl alcohol	0.29 ± 0.02bc	0.32 ± 0.03ab	0.32 ± 0.02ab	0.38 ± 0.03a	0.33 ± 0.02ab	0.25 ± 0.02c
1125	benzyl acetate	0.17 ± 0.02b	0.18 ± 0.02b	0.24 ± 0.01a	0.24 ± 0.03a	0.16 ± 0.02b	0.15 ± 0.02b
1161	*p*-creosol	0.63 ± 0.07bc	0.72 ± 0.05bc	0.92 ± 0.10b	1.53 ± 0.26a	0.42 ± 0.00cd	0.26 ± 0.03d
1166	methyl salicylate	0.13 ± 0.01b	0.14 ± 0.01b	0.16 ± 0.01b	<0.01	0.26 ± 0.01a	0.29 ± 0.05a
1172	safranal	0.06 ± 0.01b	0.06 ± 0.01b	0.08 ± 0.01ab	0.10 ± 0.02a	0.07 ± 0.01ab	0.06 ± 0.01b
1194	β-cyclocitral	0.10 ± 0.03a	0.09 ± 0.03a	<0.01	<0.01	<0.01	0.07 ± 0.01a
1212	anisaldehyde	0.12 ± 0.02a	0.11 ± 0.02a	0.13 ± 0.03a	0.13 ± 0.01a	0.14 ± 0.02a	0.12 ± 0.01a
1221	phenethyl acetate	0.07 ± 0.02ab	0.07 ± 0.01ab	0.10 ± 0.03a	0.11 ± 0.01a	0.07 ± 0.01ab	0.05 ± 0.01b
1232	cinnamaldehyde	0.05 ± 0.02a	0.05 ± 0.01a	0.07 ± 0.03a	-	-	-
1250	anise alcohol	0.32 ± 0.03a	0.28 ± 0.02a	0.30 ± 0.07a	0.29 ± 0.01a	0.37 ± 0.04a	0.38 ± 0.05a
1268	cinnamyl alcohol	0.04 ± 0.01a	0.04 ± 0.01a	0.05 ± 0.01a	0.08 ± 0.03a	0.04 ± 0.01a	-
1280	*p*-vinylguaiacol	0.13 ± 0.02a	0.15 ± 0.02a	0.16 ± 0.05a	0.17 ± 0.02a	0.16 ± 0.03a	0.15 ± 0.01a
1290	piperonal	0.04 ± 0.01a	0.04 ± 0.02a	0.07 ± 0.02a	0.06 ± 0.01a	<0.01	-
1335	*p*-hydroxybenzaldehyde	0.79 ± 0.04a	0.51 ± 0.06ab	0.29 ± 0.24b	0.44 ± 0.19b	0.92 ± 0.02a	1.05 ± 0.08a
1373	vanillin	79.25 ± 4.40a	78.27 ± 6.82a	72.24 ± 13.94a	63.14 ± 13.61a	79.70 ± 3.37a	82.39 ± 13.36a
1380	α-copaene	0.22 ± 0.03a	0.21 ± 0.02a	0.27 ± 0.07a	0.29 ± 0.04a	0.25 ± 0.01a	0.19 ± 0.07a
1418	α-santalene	-	-	-	0.14 ± 0.09a	0.13 ± 0.01a	0.22 ± 0.07a
1428	methylvanillin	0.19 ± 0.03a	0.19 ± 0.04a	0.22 ± 0.06a	0.29 ± 0.05a	-	<0.01
1465	β-ionone	0.08 ± 0.04a	0.09 ± 0.02a	0.10 ± 0.04a	0.12 ± 0.02a	<0.01	0.16 ± 0.07a
1474	α-amorphene	0.08 ± 0.02b	0.06 ± 0.01b	0.08 ± 0.01b	0.12 ± 0.02a	0.09 ± 0.01b	0.09 ± 0.00b
1498	α-muurolene	0.14 ± 0.04b	0.11 ± 0.03b	0.11 ± 0.04b	0.22 ± 0.01a	0.16 ± 0.02ab	-
1517	δ-cadinene	-	-	0.04 ± 0.01b	0.09 ± 0.01a	0.06 ± 0.01b	0.05 ± 0.01b
	unknown	7.73	10.48	13.79	7.08	10.48	8.68
	total	89.00	88.40	84.11	78.83	87.83	89.93

^A^ Retention indices, using paraffin (C5–C25) as references. ^B^ Tentatively identification of components based on GC-MS library (Wiley 7n). ^C^ Relative percentages from GC-FID, values are means ± SD of triplicates. Different letters within the same line denote significant difference in Tukey’s multiple test (p < 0.05). ^D^ undectable. ^E^ K1- immersion in hot water at 65 °C for 1 min, repeated 3 times. ^F^ K2- immersion in hot water at 65 °C for 3 min. ^G^ K3- immersion in hot water at 70 °C for 30 s, repeated 3 times. ^H^ K4- immersion in hot water at 80 °C for 10 s, repeated 3 times. ^I^ S1- sweating 40 °C. ^J^ S2- sweating 45.

**Table 4:** Comparisons of volatile compounds and their relative percentage from vanilla pods supplemented with different microorganism treatment.

RI ^A^	Compounds ^B^	Relative Percentage ^C^ (%)				
		Control	Microorganism Treatment			
		Ctrl ^E^	Y1 ^F^	Y2 ^G^	Y3 ^H^	B ^I^
799	furfural	0.41 ± 0.19a	0.75 ± 0.18a	0.66 ± 0.06a	0.44 ± 0.10a	0.56 ± 0.13a
928	benzaldehyde	0.31 ± 0.17ab	0.46 ± 0.11a	0.34 ± 0.04ab	0.23 ± 0.09ab	0.15 ± 0.03b
975	2-pentylfuran	- ^D^	0.72 ± 0.20a	0.51 ± 0.04ab	-	0.10 ± 0.02b
999	benzyl alcohol	0.74 ± 0.38bc	1.86 ± 0.25a	1.94 ± 0.07ab	1.41 ± 0.47ab	0.36 ± 0.04c
1007	2-ethyl-1-hexanol	-	-	-	-	0.46 ± 0.09
1016	limonene	0.11 ± 0.01b	0.27 ± 0.03a	0.16 ± 0.06b	0.09 ± 0.03b	0.10 ± 0.00b
1030	acetophenone	-	<0.01	<0.01	<0.01	<0.01
1035	3,5-octadien-2-one	0.08 ± 0.04b	0.13 ± 0.03ab	0.12 ± 0.02ab	0.08 ± 0.03b	0.18 ± 0.03a
1043	*p*-cresol	0.41 ± 0.22b	1.06 ± 0.31a	0.74 ± 0.02ab	0.60 ± 0.12ab	0.63 ± 0.05ab
1049	1-octanol	-	-	0.13 ± 0.01	0.09 ± 0.03	-
1056	guaiacol	2.01 ± 0.91a	2.08 ± 0.39a	2.00 ± 0.08a	1.68 ± 0.43a	0.80 ± 0.07a
1061	tetramethylpyrazine	0.05 ± 0.02a	-	0.06 ± 0.00a	0.04 ± 0.01a	-
1074	nonanal	0.20 ± 0.18b	0.16 ± 0.02b	0.13 ± 0.00b	0.12 ± 0.03b	0.54 ± 0.07a
1080	phenethyl alcohol	0.22 ± 0.07c	0.35 ± 0.04ab	0.33 ± 0.01bc	0.24 ± 0.05bc	0.41 ± 0.04a
1125	benzyl acetate	0.12 ± 0.03b	0.29 ± 0.05a	0.25 ± 0.02ab	0.17 ± 0.02b	0.25 ± 0.03ab
1161	*p*-creosol	0.38 ± 0.13bc	0.68 ± 0.08a	0.62 ± 0.02a	0.47 ± 0.10ab	0.24 ± 0.01c
1166	methyl salicylate	0.17 ± 0.07b	0.37 ± 0.06a	0.23 ± 0.00b	0.16 ± 0.04b	0.15 ± 0.00b
1172	safranal	0.05 ± 0.02b	-	0.09 ± 0.00a	0.07 ± 0.02ab	0.09 ± 0.01a
1177	decanal	0.06 ± 0.04	0.11 ± 0.02	-	-	-
1212	anisaldehyde	0.08 ± 0.03a	0.09 ± 0.01a	0.08 ± 0.01a	0.07 ± 0.00a	0.11 ± 0.03a
1221	phenethyl acetate	0.05 ± 0.03a	0.05 ± 0.01a	0.05 ± 0.00a	0.04 ± 0.00a	-
1250	anise alcohol	0.25 ± 0.07a	0.25 ± 0.03a	0.24 ± 0.00a	0.26 ± 0.04a	0.26 ± 0.06a
1268	cinnamic alcohol	0.04 ± 0.03a	0.09 ± 0.03a	0.05 ± 0.00a	0.04 ± 0.00a	-
1280	*p*-vinylguaiacol	0.10 ± 0.03b	0.51 ± 0.17a	0.12 ± 0.00b	0.12 ± 0.01b	0.30 ± 0.04ab
1290	piperonal	-	-	-	-	0.15 ± 0.03
1335	*p*-hydroxybenzaldehyde	0.05 ± 0.03b	-	0.50 ± 0.05a	0.35 ± 0.26ab	0.58 ± 0.11a
1373	vanillin	73.85 ± 11.51a	66.13 ± 12.39a	71.14 ± 3.06a	78.10 ± 24.03a	83.69 ± 14.12a
1380	α-copaene	0.15 ± 0.05c	0.43 ± 0.08a	0.39 ± 0.03ab	0.27 ± 0.04bc	0.21 ± 0.04c
1418	α-santalene	-	0.10 ± 0.02	-	-	-
1428	methylvanillin	0.13 ± 0.10a	0.15 ± 0.07a	0.28 ± 0.05a	0.21 ± 0.03a	-
1441	α-bergamotene	0.09 ± 0.03	0.10 ± 0.00	-	-	-
1446	acetovanillone	0.08 ± 0.03b	0.13 ± 0.04b	0.10 ± 0.00b	0.09 ± 0.01b	0.52 ± 0.04a
1474	α-amorphene	0.08 ± 0.06b	0.20 ± 0.02a	0.15 ± 0.06ab	0.10 ± 0.02b	-
1498	α-muurolene	0.12 ± 0.02a	0.16 ± 0.10a	0.14 ± 0.03a	0.12 ± 0.03a	-
1517	δ-cadinene	0.05 ± 0.02	0.08 ± 0.03	-	-	-
	unknown	3.78	7.55	5.59	3.85	1.01
	total	83.23	85.76	87.35	89.48	91.84

^A^ Retention indices, using paraffin (C_5_-C_25_) as references. ^B^ Tentatively identification of components based on GC-MS library (Wiley 7n). ^C^ Relative percentages from GC-FID, values are means ± SD of triplicates Different letters within the same line denote significant difference in Tukey’s multiple test (*p* < 0.05). ^D^ undectable. ^E^ Ctrl- Control group. ^F^ Y1- *D. bruxellensis.* ^G^ Y2- *S. crataegensis.* ^H^ Y3- *C. cacaoi*. **^I^**B- *B. subtilis.*

**Table 5:** Volatile compounds and their relative percentage in vanilla cultivated in different regions of Taiwan.

RI ^A^	Compounds ^B^	Relative Percentage ^C^ (%)						
		A ^E^	B ^F^	C ^G^	D ^H^	E ^I^	F ^J^	G ^K^
799	furfural	0.31 ± 0.01ab	0.18 ± 0.05ab	0.12 ± 0.03b	0.15 ± 0.05ab	0.25 ± 0.01ab	0.13 ± 0.01b	0.37 ± 0.20a
928	benzaldehyde	0.33 ± 0.01a	0.08 ± 0.01d	0.19 ± 0.02c	0.17 ± 0.04c	0.20 ± 0.01bc	0.20 ± 0.06bc	0.28 ± 0.00ab
975	2-pentylfuran	0.24 ± 0.02ab	0.10 ± 0.01b	0.16 ± 0.03ab	0.15 ± 0.03ab	0.16 ± 0.01ab	0.14 ± 0.04ab	0.31 ± 0.14a
999	benzyl alcohol	0.85 ± 0.04ab	0.33 ± 0.04b	0.39 ± 0.23b	0.39 ± 0.15b	0.49 ± 0.02ab	0.40 ± 0.09b	1.00 ± 0.37a
1006	salicylaldehyde	0.41 ± 0.04a	- ^D^	0.32 ± 0.02b	-	-	0.16 ± 0.04c	0.24 ± 0.00d
1014	limonene	0.12 ± 0.04a	0.09 ± 0.02a	0.10 ± 0.01a	0.12 ± 0.01a	0.11 ± 0.00a	0.13 ± 0.04a	0.07 ± 0.05a
1030	acetophenone	<0.01	0.05 ± 0.00a	0.06 ± 0.01a	0.05 ± 0.01a	0.05 ± 0.00a	0.04 ± 0.01a	0.04 ± 0.01a
1035	3,5-octadien-2-one	0.10 ± 0.03a	0.07 ± 0.01a	0.07 ± 0.01a	0.08 ± 0.02a	0.10 ± 0.01a	0.08 ± 0.02a	0.08 ± 0.01a
1043	*p*-cresol	0.72 ± 0.02a	0.25 ± 0.03b	0.22 ± 0.02ab	0.60 ± 0.07b	0.28 ± 0.02b	0.24 ± 0.03b	0.74 ± 0.35a
1056	guaiacol	5.25 ± 0.20a	2.41 ± 0.14b	2.22 ± 0.55b	2.87 ± 0.11b	2.16 ± 0.07bc	2.78 ± 0.16b	2.55 ± 0.74b
1074	nonanal	0.32 ± 0.06a	0.10 ± 0.00d	0.22 ± 0.00abc	0.27 ± 0.05bc	0.18 ± 0.01bcd	0.14 ± 0.03cd	0.13 ± 0.07cd
1080	phenethyl alcohol	0.61 ± 0.03a	0.24 ± 0.03b	0.28 ± 0.01b	0.21 ± 0.03b	0.24 ± 0.01b	0.25 ± 0.03b	0.28 ± 0.04b
1125	benzyl acetate	0.10 ± 0.02ab	0.06 ± 0.01b	0.08 ± 0.01b	0.10 ± 0.03ab	0.07 ± 0.00b	0.07 ± 0.01b	0.15 ± 0.04a
1161	*p*-creosol	0.95 ± 0.04a	0.37 ± 0.03bc	0.32 ± 0.02c	0.47 ± 0.05bc	0.32 ± 0.02c	0.39 ± 0.03bc	0.54 ± 0.16b
1166	methyl salicylate	0.54 ± 0.01a	0.12 ± 0.01b	0.14 ± 0.01b	0.14 ± 0.01b	0.12 ± 0.01b	0.13 ± 0.01b	0.13 ± 0.00b
1172	safranal	0.10 ± 0.01b	0.07 ± 0.02bc	0.07 ± 0.01bc	0.14 ± 0.02a	0.06 ± 0.00c	0.06 ± 0.01c	0.06 ± 0.01cd
1212	anisaldehyde	0.16 ± 0.01ab	0.12 ± 0.01c	0.19 ± 0.00a	0.11 ± 0.02c	0.12 ± 0.01c	0.13 ± 0.01bc	0.12 ± 0.02c
1221	phenethyl acetate	0.07 ± 0.01a	0.04 ± 0.01a	0.05 ± 0.00a	0.06 ± 0.01a	0.05 ± 0.02a	0.04 ± 0.00a	0.06 ± 0.02a
1250	anise alcohol	0.24 ± 0.01a	0.15 ± 0.01a	0.20 ± 0.11a	0.22 ± 0.06a	0.18 ± 0.01a	0.19 ± 0.02a	0.28 ± 0.10a
1268	cinnamic alcohol	0.28 ± 0.10a	0.05 ± 0.02b	0.04 ± 0.01b	0.05 ± 0.01b	<0.01	-	<0.01
1280	*p*-vinylguaiacol	0.21 ± 0.01a	0.17 ± 0.01abc	0.15 ± 0.00abcd	0.18 ± 0.05ab	0.07 ± 0.00d	0.09 ± 0.04cd	0.10 ± 0.04bcd
1290	piperonal	0.06 ± 0.01ab	0.04 ± 0.01b	0.06 ± 0.01ab	0.09 ± 0.03a	0.05 ± 0.00ab	0.05 ± 0.02ab	0.03 ± 0.02b
1335	*p*-hydroxybenzaldehyde	0.39 ± 0.04b	0.44 ± 0.07b	0.84 ± 0.07a	0.85 ± 0.09a	0.67 ± 0.08ab	0.76 ± 0.16a	0.74 ± 0.14a
1373	vanillin	67.95 ± 11.71a	83.11 ± 5.37a	78.43 ± 11.65a	77.04 ± 4.81a	82.36 ± 1.49a	83.32 ± 16.80a	78.69 ± 9.03a
1380	α-copaene	0.17 ± 0.02b	0.15 ± 0.01b	0.21 ± 0.03ab	0.22 ± 0.01ab	0.25 ± 0.02a	0.17 ± 0.04b	0.22 ± 0.04ab
1418	α-santalene	0.32 ± 0.01a	0.23 ± 0.02ab	0.29 ± 0.01ab	0.34 ± 0.07a	0.26 ± 0.03ab	0.25 ± 0.03ab	0.20 ± 0.06a
1428	methylvanillin	0.20 ± 0.00a	<0.01	0.21 ± 0.02a	<0.01	<0.01	0.21 ± 0.02a	<0.01
1446	acetovanillone	-	0.09 ± 0.01b	<0.01	0.23 ± 0.13a	0.09 ± 0.00b	0.09 ± 0.01b	0.09 ± 0.02b
1474	α-amorphene	<0.01	-	0.08 ± 0.01a	0.14 ± 0.07a	0.09 ± 0.02a	-	-
1498	α-muurolene	0.23 ± 0.01ab	-	0.17 ± 0.01b	0.30 ± 0.08a	0.16 ± 0.02b	<0.01	-
1517	δ-cadinene	0.09 ± 0.01	-	-	0.14 ± 0.09	-	-	-
	unknown	3.88	6.76	10.09	11.54	8.71	6.29	9.48
	total	85.21	91.39	88.22	86.18	90.01	92.17	89.03

^A^ Retention indices, using paraffin (C_5_–C_25_) as references. ^B^ Tentatively identification of components based on GC-MS library (Wiley 7n). ^C^ Relative percentages from GC-FID, values are means ± SD of triplicates. Different letters within the same line denote significant difference in Tukey’s multiple test (*p* < 0.05). ^D^ undectable. ^E^ A- Nantou Mingjian. ^F^ B- Taitung Station, ^G^ C- Taoyuan Station. ^H^ D- Yunlin Sihu. ^I^ E- Hsinchu Xinpu. ^J^ F- Taoyuan Longtan;.^K^ G- Taoyuan Xinwu.

**Table 6:** All vanilla pod samples used in this study.

Sample Conditions	Sample Name
Normal process	control
Different harvest maturity	pollinated at 34 weeks prior to harvest
	pollinated at 38 weeks prior to harvest
	pollinated at 42 weeks prior to harvest
Different curing conditions	sweating at 40 °C
	sweating at 45 °C
	immersion in hot water at 65 °C three times for 1 min each
	immersion in hot water at 65 °C for 3 min
	immersion in hot water at 70 °C three times for 30 s each
	immersion in hot water at 80 °C three times for 10 s each
Microorganism treatment	*Dekkera bruxellensis*
	*Saccharomycopsis crataegensis*
	*Candida cacaoi*
	*Bacillus subtilis*
Different region in Taiwan	Nantou Mingjian
	Taitung Station
	Taoyuan Station
	Yunlin Sihu
	Hsinchu Xinpu
	Taoyuan Xinwu
	Taoyuan Longtan

## Data Availability

Data sharing not applicable. No new data were created or analyzed in this study. Data sharing is not applicable to this article.

## Sample Availability

Samples of the compounds are not available from the authors.

## References

[1] Gallage N.J., Moller B.L. (2015). Vanillin-bioconversion and bioengineering of the most popular plant flavor and its de novo biosynthesis in the vanilla orchid. Mol. Plant.

[2] Bythrow J.D. (2005). Vanilla as a Medicinal Plant. Semin. Integr. Med..

[3] Anuradha K., Shyamala B.N., Naidu M.M. (2013). Vanilla—Its science of cultivation, curing, chemistry, and nutraceutical properties. Crit. Rev. Food Sci. Nutr..

[4] Korthou H., Verpoorte R. (2007). Vanilla. Flavours and Fragrances.

[5] Ramachandra Rao S., Ravishankar G.A. (2000). Vanilla flavour: Production by conventional and biotechnological routes. J. Sci. Food Agric..

[6] Alexander C., Venkatramanan A. (2012). Analytic Approximations for Multi-Asset Option Pricing. Math. Financ..

[7] Anuradha K., Naidu M.M., Manohar R.S., Indiramma A.R. (2010). Effect of vanilla extract on radical scavenging activity in biscuits. Flavour Fragr. J..

[8] Aguirre-Alonso R.O., Morales-Guillermo M., Salgado-Cervantes M.A., Robles-Olvera V.J., García-Alvarado M.A., Rodríguez-Jimenes G.C. (2019). Effect of process variables of spray drying employing heat pump and nitrogen on aromatic compound yield in powders obtained from vanilla (Vanilla planifolia Andrews) ethanolic extract. Dry. Technol..

[9] Wilde A.S., Frandsen H.L., Fromberg A., Smedsgaard J., Greule M. (2019). Isotopic characterization of vanillin ex glucose by GC-IRMS-New challenge for natural vanilla flavour authentication?. Food Control..

[10] Brunschwig C., Rochard S., Pierrat A., Rouger A., Senger-Emonnot P., George G., Raharivelomanana P. (2016). Volatile composition and sensory properties of Vanilla x tahitensis bring new insights for vanilla quality control. J. Sci. Food Agric..

[11] Gillette M., Hoffman P. (1992). Vanilla extract. Encycl. Food Sci. Technol..

[12] Takahashi M., Inai Y., Miyazawa N., Kurobayashi Y., Fujita A. (2013). Identification of the key odorants in Tahitian cured vanilla beans (Vanilla tahitensis) by GC-MS and an aroma extract dilution analysis. Biosci. Biotechnol. Biochem..

[13] Brunschwig C., Senger-Emonnot P., Aubanel M.L., Pierrat A., George G., Rochard S., Raharivelomanana P. (2012). Odor-active compounds of Tahitian vanilla flavor. Food Res. Int..

[14] Hassan S., Araceli P.-S., Denis B., los Ángeles V.-V.M.d., Mayra N.-G., Delfino R.-L. (2016). Identification of volatile compounds in cured Mexican vanilla (Vanilla planifoliaG. Jackson) beans using headspace solid-phase microextraction with gas chromatography-mass spectrometry. Fruits.

[15] Liang H., Lu J., Dai Y., Li X., Guo S., Li Q. (2014). Analysis of the Volatile Components in the Fruits of Vanilla planifoli Andrews by HS-SPME Combined with GC-MS. Med. Plant.

[16] Xu M., Jin Z., Gu Z., Rao J., Chen B. (2020). Changes in odor characteristics of pulse protein isolates from germinated chickpea, lentil, and yellow pea: Role of lipoxygenase and free radicals. Food Chem..

[17] Pérez-Silva A., Odoux E., Brat P., Ribeyre F., Rodriguez-Jimenes G., Robles-Olvera V., García-Alvarado M.A., Günata Z. (2006). GC–MS and GC–olfactometry analysis of aroma compounds in a representative organic aroma extract from cured vanilla (Vanilla planifolia G. Jackson) beans. Food Chem..

[18] Mariezcurrena M.D., Zavaleta H.A., Waliszewski K.N., Snchez V. (2008). The effect of killing conditions on the structural changes in vanilla (Vanilla planifolia, Andrews) pods during the curing process. Int. J. Food Sci. Technol..

[19] Pardio V.T., Flores A., Lopez K.M., Martinez D.I., Marquez O., Waliszewski K.N. (2018). Effect of endogenous and exogenous enzymatic treatment of green vanilla beans on extraction of vanillin and main aromatic compounds. J. Food Sci. Technol..

[20] Roling W.F., Kerler J., Braster M., Apriyantono A., Stam H., van Verseveld H.W. (2001). Microorganisms with a taste for vanilla: Microbial ecology of traditional Indonesian vanilla curing. Appl. Environ. Microbiol..

[21] Chen Y., Gu F., Li J., He S., Xu F., Fang Y. (2015). Involvement of Colonizing Bacillus Isolates in Glucovanillin Hydrolysis during the Curing of Vanilla planifolia Andrews. Appl. Environ. Microbiol..

[22] Gu F., Chen Y., Fang Y., Wu G., Tan L. (2015). Contribution of Bacillus Isolates to the Flavor Profiles of Vanilla Beans Assessed through Aroma Analysis and Chemometrics. Molecules.

[23] Komes D., Ulrich D., Lovric T. (2005). Characterization of odor-active compounds in Croatian Rhine Riesling wine, subregion Zagorje. Eur. Food Res. Technol..

[24] Schifferdecker A.J., Dashko S., Ishchuk O.P., Piskur J. (2014). The wine and beer yeast Dekkera bruxellensis. Yeast.

[25] Niu Y., Zhang X., Xiao Z., Song S., Eric K., Jia C., Yu H., Zhu J. (2011). Characterization of odor-active compounds of various cherry wines by gas chromatography-mass spectrometry, gas chromatography-olfactometry and their correlation with sensory attributes. J. Chromatogr. B Analyt. Technol. Biomed. Life Sci..

[26] Takahashi M., Inai Y., Miyazawa N., Kurobayashi Y., Fujita A. (2013). Key odorants in cured Madagascar vanilla beans (Vanilla planiforia) of differing bean quality. Biosci. Biotechnol. Biochem..

[27] Januszewska R., Giret E., Clement F., Van Leuven I., Goncalves C., Vladislavleva E., Pradal P., Nabo R., Landuyt A., D'Heer G. (2020). Impact of vanilla origins on sensory characteristics of chocolate. Food Res. Int..

[28] Kovats E. (1958). Gas chromatographic characterization of organic compounds. I. Retention indexes of aliphatic halides, alcohols, aldehydes, and ketones. Helv. Chim. Acta.

